# Wood-derived lignans magnolol and honokiol improve nutrient utilization in broilers and regulate inflammation and epithelial repair in vitro

**DOI:** 10.1016/j.psj.2026.107625

**Published:** 2026-08-15

**Authors:** Mara Heckmann, Verena Stadlbauer, Bettina Schwarzinger, Nina Neufeld, Klaus Männer, Julian Weghuber, Bernhard Blank-Landeshammer

**Affiliations:** a Josef Ressel Centre for Innovation in Bioavailability Research, Center of Excellence Food Technology and Nutrition, University of Applied Sciences Upper Austria, Stelzhamerstraße 23, 4600, Wels, Austria; b Center of Excellence Food Technology and Nutrition, University of Applied Sciences Upper Austria, Stelzhamerstraße 23, 4600, Wels, Austria; c FFoQSI GmbH–Austrian Competence Centre for Feed and Food Quality, Safety and Innovation, Technopark 1D, 3430, Tulln, Austria; d Agromed Austria GmbH, Bad Haller Straße 23, 4550, Kremsmünster, Austria; e Institute of Animal Nutrition, Freie Universität Berlin, Königin-Luise-Straße 49, 14195, Berlin, Germany

**Keywords:** Wood-based lignans, Feed supplement, Broiler performance, Magnolol, Honokiol

## Abstract

Plant-derived lignans are increasingly investigated as components of feed supplements in poultry production due to their antioxidant, anti-inflammatory and intestinal barrier-supporting properties. However, direct evidence linking lignan supplementation to improved nutrient digestibility remains limited, and the individual contributions of magnolol and honokiol to the biological effects of such additives are not yet fully understood. This study therefore examined the effects of a wood-derived lignan feed supplement containing both compounds on growth performance, nutrient utilization and intestinal health in broiler chickens, alongside targeted in vitro mechanistic investigations. In a 35-day broiler feeding trial, 180 birds in 6 pens per treatment received a control diet or the lignan supplement at 0.1 g/kg feed. Supplementation increased body weight by 90.8 g and reduced feed-to-gain ratio from 1.41 to 1.32. Apparent ileal digestibility of crude protein, crude ash, calcium and phosphorus increased by 2.8 %, 3.9 %, 5.1 % and 2.2 %, respectively. Jejunal gene expression analysis revealed a significant downregulation of IL-8, while TNF-α and IL-10 remained unaffected. To further investigate underlying mechanisms, magnolol and honokiol were evaluated in intestinal epithelial and immune cell models. Transepithelial transport was documented for both lignans in the Caco-2 model, with magnolol achieving higher apparent permeability. Magnolol enhanced wound closure speed in IPEC-J2 cells, reflecting a capacity to support epithelial restitution after injury. In contrast, honokiol reduced intracellular reactive oxygen species in epithelial cells and strongly suppressed nitric oxide production in LPS-stimulated macrophages, pointing to a dominant role in redox and inflammatory signaling. Both compounds inhibited NF-κB activation, though honokiol acted more broadly across TLR4-dependent and -independent pathways. These in vitro findings provide possible explanations for the in vivo effects, although their contribution to the observed responses in broilers remains to be established. Overall, magnolol and honokiol provide complementary and mechanistically distinctive activities in vitro and their combined use in a single feed supplement may therefore represent a promising strategy for improving nutrient utilization and productive performance in poultry production.

## Introduction

The gastrointestinal tract is a central interface between the external environment and the intestinal physiology of the animal. Efficient nutrient utilization therefore depends not only on digestion and absorption, but also on the maintenance of the intestinal epithelial integrity and a tightly regulated mucosal immune system (Celi et al., 2017). Disturbances in gut barrier function can promote inflammatory processes, impair nutrient utilization and reduce animal performance. In intensive livestock production, this balance can be challenged by rapid growth, high nutrient intake and environmental stressors, all of which may contribute to oxidative stress and low-grade intestinal inflammation (Mishra and Jha, 2019; Rostagno, 2020). Moreover, it is increasingly recognized that feed intake itself can induce measurable immune activation in the gut, making the control of unnecessary intestinal inflammation highly relevant for metabolic efficiency and productivity (Kogut et al., 2018).

For decades, antibiotic growth promoters (AGPs) were used to improve feed efficiency and growth performance. Their beneficial effects are now thought to be explained not only by antimicrobial activity, but also by anti-inflammatory actions within the gastrointestinal tract. By reducing intestinal inflammatory stress, AGPs can lower immune-related energy expenditure and endogenous nutrient losses, thereby improving nutrient digestibility and growth efficiency (Fernández Miyakawa et al., 2024; Rahman et al., 2022). Due to concerns regarding antimicrobial resistance, AGPs were banned in the European Union from January 1, 2006, and production uses of medically important antimicrobials were eliminated in the United States through the Veterinary Feed Directive implemented by the Food and Drug Administration (FDA) in 2017 (Castanon, 2007; Sarkar and Okafor, 2024). These developments have intensified the search for non-antibiotic feed additives that support intestinal health and performance.

Among such alternatives, plant-derived bioactive compounds received considerable attention because of their antioxidant, anti-inflammatory and barrier-supporting properties (Abdelli et al., 2021; Hashemi and Davoodi, 2011; Heckmann et al., 2025b). Within this group, lignans are of particular interest. As plant polyphenols, lignans have been associated with potential application in strategies aimed at maintaining intestinal homeostasis and metabolic efficiency (Burgberger et al., 2025). The wood-derived biphenolic neolignans magnolol and honokiol are naturally occurring in the bark of *Magnolia officinalis,* which has been used in traditional Chinese medicine for more than 2,000 years (Luo et al., 2019), and have been reported to exhibit multiple biological activities (Dai et al., 2023; Mottaghi and Abbaszadeh, 2022). Feed additives containing these wood lignans are increasingly used in animal nutrition and are typically applied at low dietary inclusion levels. In laying hens, dietary magnolol supplementation has been shown to enhance egg production and egg quality while improving intestinal morphology and mucosal barrier function (Chen et al., 2021b). Dietary honokiol has been reported to enhance antioxidant capacity, improve intestinal morphology and influence microbial metabolic activity in broiler chickens (Du et al., 2024). Another study in poultry demonstrated that supplementation with honokiol or magnolol can mitigate pathogen-induced intestinal damage, improve growth performance and modulate intestinal microbiota and immune-related gene expression (Chen et al., 2021a). While these limited studies have shown beneficial effects of magnolol and honokiol on intestinal barrier and health, a comprehensive mechanistic understanding of epithelial transport or epithelial repair is currently lacking.

In a previous study, we demonstrated that extracts prepared from a wood-based feed supplement containing magnolol and honokiol (AGROMED ROI, agromed Austria GmbH), exert anti-inflammatory effects in Tohoku Hospital Pediatrics-1 (THP-1) macrophages. More specifically, this extract significantly reduced the expression and secretion of pro-inflammatory mediators including interleukin (IL)-8, IL-6 and tumor necrosis factor alpha (TNF-α) and improved parameters related to barrier dysfunction, providing first evidence that Magnolia-derived lignans may support intestinal immune balance in a feed-related context (Heckmann et al., 2022). Building on these findings, the present study aimed to further investigate the biological effects of this wood-lignan-based feed supplement. A 35-day feeding trial in broilers was conducted to evaluate the effects on productive performance, nutrient digestibility and intestinal inflammatory status. In parallel, complementary in vitro experiments were performed with the commercially available pure compounds magnolol and honokiol to obtain mechanistic insight into their intestinal transport and their effects on oxidative stress, inflammatory signaling and epithelial restitution and wound healing. Together, these experiments were designed to clarify how the major lignans present in the tested feed supplement may contribute to intestinal health and metabolic efficiency in broiler chickens.

## Materials and methods

### Investigated feed additive and its main lignans

The investigated feed supplement AGROMED ROI MAX was obtained from agromed Austria GmbH (operating under A&P Nutrition trademark; Kremsmünster, Austria). This product sold for poultry and pigs is a solid fine granulate and contains lignocellulose, calcium carbonate, silica and zinc chloride (Heckmann et al., 2022). The analyzed content of the active compounds magnolol and honokiol is 0.73 % and 0.45 % (w/w), respectively, while the zinc content is 55.9 g/kg (analyzed by AGROLAB LUFA GmbH, Kiel, Germany). For the feeding trial, this feed supplement was incorporated into the diet in its solid form.

For cell culture experiments, the main lignans of the feed supplement, magnolol and honokiol (purity ≥ 99 %), were purchased from Extrasynthese (Genay Cedex, France).

### Broiler feeding trial

***Experimental design.*** The study was conducted as a 35-day feeding trial with 180 broiler chickens allocated to two experimental groups. Healthy male Cobb 500 one-day-old broiler chickens were obtained from a local breeder and randomly allocated to 12 floor pens with 15 birds per pen. Pens were randomly assigned to two dietary treatments (6 pens per treatment): the control group received the basal diet while the trial group received the basal diet containing the feed supplement at a dosage of 0.1 g/kg feed for 35 days, replacing an equivalent amount of silica carrier (Tixosil). To ensure homogenous distribution, 15 kg premixes were first prepared by gently mixing aliquots of the basal diet with the total required amount of Tixosil (control group) or the feed supplement (trial group). These premixes were subsequently incorporated into the corresponding final feed batches and thoroughly mixed. The mixtures were prepared in two twin-shaft horizontal mixers using two parallel, synchronized shafts rotating in opposite directions within the optimum filling range of 60 % over a period of 5 min*.* A two-phase feeding program was used consisting of two mash diets: a starter diet from day 0-14 and a grower diet from day 15-35, both formulated to meet the nutrient requirements for broilers according to the Society of Nutrition Physiology (GfE) (Ausschuss f. Bedarfsnormen d. Gesellschaft f. Ernährungsphysiologie, 1999). The ingredient composition and calculated contents of both diets are shown in Table 1, Table 2, respectively.

**Table 1:** Compositions of the starter and grower diets in the broiler feeding trial (%).

Ingredient	Starter diet	Grower diet
Soybean meal (crude protein: 49 %)	33.31	28.06
Maize	33.16	31.74
Wheat	25.20	30.00
Soybean oil	4.00	5.30
Limestone	1.48	1.12
Monocalcium-phosphate	1.38	1.03
Minerals and vitamins1)	1.20	1.20
DL-Methionine	0.16	0.20
L-Lysine HCL	0.10	0.24
Tixosil2) or feed supplement	0.01	0.01
Celite3)	-	1.00
L-Threonine	-	0.10

1) Contents per kg: 600,000 I.U. Vit. A (acetate); 120,000 I.U. Vit. D_3_; 1500 mg vit. E (α-tocopherol acetate); 200 mg Vit. K_3_ (menadione sodium bisulfite); 250 mg Vit. B_1_ (mononitrate); 420 mg Vit. B_2_ (cryst. Riboflavin); 300 mg Vit. B_6_ (pyridoxin-HCl); 1500 μg Vit. B_12_; 3000 mg niacin (niacinamide); 12500 μg biotin (commercial, feed grade); 100 mg folic acid (cryst., commercial, feed grade); 1000 mg pantothenic acid (Ca D-pantothenate), 60000 mg choline (chloride); 5000 mg iron (iron carbonate); 5000 mg zinc (zinc sulfate); 6000 mg manganese (manganous oxide); 1000 mg copper (copper oxide); 45 mg iodine (calcium-iodate); 20 mg selenium (sodium-selenite); 140 g sodium (sodium chloride); 55 g magnesium (magnesium sulfate); carrier: wheat semolina (38 %).

2) Tixosil (> 97 % silicon dioxide) was included in the control diet and was replaced by the test substance in the treatment diet.

3) Indigestible marker for AID (>85 % silica).

**Table 2:** Calculated contents of the starter and grower diets in the broiler feeding trial.

Ingredient	Starter diet	Grower diet
AMEn1) [MJ/kg]	12.65	13.00
Crude protein [g/kg]	226.00	206.90
Lysine [g/kg]	12.90	12.50
Methionine [g/kg]	5.00	5.10
Methionine/cysteine [g/kg]	8.90	8.70
Tryptophan [g/kg]	2.30	8.70
Threonine [g/kg]	8.70	7.10
Valine [g/kg]	1.00	-
Arginine [g/kg]	1.41	-
Crude fiber [g/kg]	24.80	21.10
Crude fat [g/kg]	73.80	75.60
Starch [g/kg]	359.10	410.64
Sugars [g/kg]	42.60	39.55
Calcium [g/kg]	9.00	7.00
Total phosphorus [g/kg]	7.20	6.00
Sodium [g/kg]	1.70	1.90

1) Nitrogen-corrected apparent metabolizable energy (AMEn), estimated according to equation of WPSA 1984 (by using crude nutrients).

Stocking density corresponded to 0.3 m² per bird. This relatively low stocking density was intentionally chosen to minimize environmental and management-related confounding factors and to provide standardized housing conditions for evaluating the effects of the dietary treatment. Pens were bedded with chopped giant miscanthus (*Miscanthus* × *giganteus*) grass as litter material. Birds had ad libitum access to feed and water. Environmental conditions were controlled and monitored. House temperature was gradually reduced from 33°C at day 0 to 23°C at day 35. Relative humidity was maintained between 50 and 60 %. Lighting was provided according to standard husbandry practices, and forced ventilation ensured air speeds between 0.3 and 0.7 m/s from day 19 onward.

The experimental unit for performance parameters was the pen, with six pens per treatment. At the end of the feeding trial, individual birds were selected for terminal sampling based on body weight (BW), with birds closest to the respective treatment mean selected for subsequent digestibility and jejunal gene expression analyses.

***Determination of growth performance and European Poultry Efficiency Factor (EPEF).*** Performance parameters were recorded on a pen basis. Body weight was measured weekly and expressed as mean body weight per pen. Feed intake, BW gain, and feed conversion ratio (FCR) were calculated weekly for each pen. Birds were inspected twice daily for signs of disease, behavioral changes, or other abnormalities. Mortality was recorded with the day and cause of death.

Feed intake per bird was calculated from the total feed consumed per pen, corrected for residual feed and adjusted for mortality using bird-days (i.e., birds that died or were culled contributed only for the number of days they were present in the pen). The FCR was calculated as the ratio of mortality-adjusted feed intake and BW gain per bird for each period. The European Poultry Efficiency Factor (EPEF) was calculated as:

$$EPEF=\;\frac{\left(average\;daily\;gain\;\left[g\right]*survival\;rate\;\left[\%\right]\right)}{\left(FCR*10\right)}$$

***Determination of Apparent Ileal Digestibility (AID).*** To determine apparent ileal digestibility (AID) at day 35, acid-insoluble ash (Celite, >85 % silica) was added to the grower diet (day 15-35) at 10 g/kg as an indigestible marker (Table 1). For AID of crude protein, crude fat, crude ash, calcium and phosphorus, selected birds were sacrificed by stunning followed by exsanguination. Ileal digesta was collected from the posterior half of the ileum (Meckel’s diverticulum to 2 cm cranial to the ileocaecal junction). 24 birds per treatment were selected based on BW, with birds closest to the respective treatment mean chosen for sampling. Ileal digesta were collected from these birds and pooled in groups of three to obtain sufficient material for chemical analysis, resulting in eight pooled digesta samples per treatment. Pooled, freeze-dried ileal samples were ground to pass a 0.25 mm screen and subsequently analyzed according to the official methods of the Association of German Agricultural Analytic and Research Institutes (VDLUFA) (Verband Deutscher Landwirtschaftlicher Untersuchungs- und Forschungsanstalten VDLUFA 1993): crude protein (VDLUFA III 4.1.1, modified for macro-N determination; vario Max CN), crude fat (VDLUFA III 5.1.1), crude ash (VDLUFA III 8.1), calcium (VDLUFA VII 2.2.2.6) and phosphorus (VDLUFA VII 2.2.2.6).

AID was calculated as:

$$AID\;\left[\%\right]=100-\left(\frac{marker\;in\;feed\;\left[\%\right]}{marker\;in\;ileum\;\left[\%\right]}*\;\frac{nutrient\;in\;ileum\;\left[\%\right]}{nutrient\;in\;feed\;\left[\%\right]}\right)*100$$

***Jejunal gene expression analysis.*** At day 35, 24 birds were selected based on BW, with birds closest to the respective treatment mean selected for tissue sampling. Birds were sacrificed by stunning followed by exsanguination. The gastrointestinal tract was excised and a segment from the mid-jejunum was collected for analysis of cytokine gene expression (IL-6, IL-8, IL-10 and TNF-α). RNA isolation and quantitative PCR were performed as described previously (Duangnumsawang et al., 2022). Beta-actin (*ACTB*), glycerinaldehyd-3-phosphat dehydrogenase (*GAPDH*) and beta-2-microglobulin (*B2M*) served as housekeeping genes (Li et al., 2005). Oligonucleotide sequences of all primers are shown in Table 3. Due to limitations in RNA quality, several samples were excluded, resulting in 12 birds from the control group and 16 birds from the trial group.

**Table 3:** Oligonucleotide sequences of primers used for gene expression analysis in broiler jejunum.

Protein	Gene	Forward primer sequence (5′→3′)	Reverse primer sequence (5′→3′)	Accession number
β-Actin	*ACTB*	GAGAAATTGTGCGTGACATCA	CCTGAACCTCTCATTGCCA	L08165
GAPDH	*GAPDH*	GGTGGTGCTAAGCGTGTTA	CCCTCCACAATGCCAA	X01578
Beta-2-Microglobulin	*B2M*	AAGGAGCCGCAGGTCTAC	CTTGCTCTTTGCCGTCATAC	Z48922
IL-8	*CXCL8*	ATCTTCCACCTTCCACATCG	AGTGGCTTCCAAGGGATCTT	HM179639
TNF-α	*TNF*	CTCGTTGGTGTGGGACGAC	CGGCGGCGTATACGAAGTA	MF000729
IL-6	*IL6*	CTGCAGGACGAGATGTGCAA	AGGTCTGAAAGGCGAACAGG	AB302327
IL-10	*IL10*	CAGAGCTCCCATCTCTCTGG	GACGTGGTTACGGGAAGAAA	NM_001004414

### Cell culture and differentiation

Intestinal porcine epithelial cells-jejunum 2 (IPEC-J2) (ACC 701) and human *Cancer coli* 2 (Caco-2) cells (ACC 169) were purchased from DSMZ (Braunschweig, Germany). Radial Abelson Wirus-induced cell line 264.7 (RAW 264.7) (TIB-71) was obtained from ATCC (Manassas, VA). The cells were maintained in Dulbecco’s Modified Eagle Medium (DMEM; IPEC-J2 and RAW 264.7) or Minimum Essential Medium Eagle (MEM Eagle; Caco-2) supplemented with 10 % fetal bovine serum (FBS) and 100 U/mL penicillin/100 μg/mL streptomycin (all PAN-Biotech, Aidenbach, Germany). The HEK-Blue human toll-like receptor 4 (hTLR4) reporter cell line, which stably co-expresses a nuclear factor kappa B (NF-κB)-inducible secreted embryonic alkaline phosphatase (SEAP) reporter gene and the hTLR4 gene, was obtained from InvivoGen (San Diego, CA), along with its corresponding parental cell line, HEK-Blue null2, which only expresses the SEAP reporter gene. These cell lines were cultured in DMEM supplemented with 10 % heat-inactivated FBS, 100 U/mL penicillin, 100 μg/mL streptomycin, and 100 μg/mL Normocin™ (InvivoGen). Additionally, selective antibiotics 1X HEK-Blue Selection were used for the HEK-Blue hTLR4 cell line, and 100 μg/mL Zeocin for the HEK-Blue null2 cell line (both from InvivoGen).

Caco-2 cells were differentiated as described previously (Haider et al., 2025) by using DMEM supplemented with 100 U/mL penicillin/100 μg/mL streptomycin, 0.1 % MITO+ Serum Extender (Corning Inc., Corning, NY) and 5 mM butyric acid (Sigma Aldrich, St. Louis, MO). This medium was applied to the cells on the day after seeding and refreshed on day 3 until complete differentiation on day four of the experiments. All cell lines were grown at 37°C in a humidified atmosphere (≥ 95 %) with 5 % CO_2_.

### Intestinal transport analysis in vitro

In vitro intestinal transport of magnolol and honokiol was analyzed by a cell culture insert‐based assay with Caco-2 cells. In detail, 5.5 × 10^5^ Caco-2 cells were seeded per insert (ThinCert, 0.336 cm², 0.4 μm pore diameter, Greiner-Bio One, Kremsmünster, Austria) to reach confluence on the next day. The cells were further maintained in full growth medium which was exchanged every 2-3 days. Trans-epithelial electrical resistance (TEER) values were measured before each medium exchange during differentiation with a Millicell‐ERS‐2 Voltohmmeter (Merck, Darmstadt, Germany). When the values reached levels higher than 1000 Ωcm² 5 days after seeding, the assay was started. Differentiated cells were washed once with PBS. Magnolol and honokiol were diluted in fasted state stimulated intestinal fluid (FaSSIF) medium (Ilardia-Arana et al., 2006) to 100 µM and added to the apical side of the cell monolayer (300 µL/insert). After 4 h of incubation, apical and basolateral samples were collected for LC-MS analysis. The apparent permeability coefficient (P_app_) values were calculated using the following formula:

$$P_{app}\;\left[cm/s\right]=\;\frac{V_{\left(basolateral\;compartment\right)}\left[ml\right]}{insert\;area\;\left[cm^{2}\right]*incubation\;time\left[s\right]}*\frac{c_{\left(basolateral\;compartment\right)}\left[\mu g/ml\right]}{c_{\left(initial\right)}\left[\mu g/ml\right]}$$

### LC-MS analysis

LC-MS analysis was carried out using a Vanquish Flex UHPLC system as described previously (Blank-Landeshammer et al., 2025). The system was equipped with a built-in degasser, a binary pump, autosampler and heated column-compartment which was coupled to an ISQ-EC mass spectrometer via a HESI ion-source (all Thermo Fisher Scientific, Waltham, MA). Chromatographic separation was achieved using an Accucore C18 column (150 mm × 2.1 mm inner diameter, 2.6 μm particle size, Thermo Fisher Scientific) with H_2_O + 5 mM ammonium formate, pH 3.65 as solvent A and 90 % acetonitrile + 5 mM ammonium formate, pH 3.65 as solvent B at a flow rate of 0.5 mL/min. Injection volume was 4 μL and gradient elution was performed by starting with 5 % solvent B, followed by a hold time of 1 min and then ramping the composition to 99.9 % B within 5 min. After a hold time of 1 min solvent B was reduced to 5 % again and an equilibration time of 5 min was used between runs. The column compartment was heated to 30°C, vaporizer temperature of the ion source was set to 375°C and the ion transfer tube was kept at 350°C. The ESI source was operated in negative ionization mode with −3.000 V and the mass of 265.2 m/z (magnolol [M-H]^−^) and honokiol [M-H]^−^) was monitored with a scan width of 0.2 amu. In addition, 247.1 m/z (magnolol) and 224.1 m/z (honokiol) as the most prominent fragment ions of the respective lignans were monitored simultaneously with a source CID voltage of 50. The resulting total scan time was 300 ms. Quantification was performed by external calibration using 7 standard dilutions in a concentration range from 10 to 1000 ng/mL with a linear regression model (R^2^ > 0.99).

### Cell migration analysis

Investigation of cell migration was performed as previously described with slight modifications (Heckmann et al., 2025b). IPEC-J2 cells were seeded in 24‐well imaging plates (MoBiTec, Göttingen, Germany) at 2.5 × 10^5^ cells per well and grown overnight. Then, a straight scratch was generated with constant pressure, speed and angle using a 100 μL plastic pipette tip. Excess debris was removed by washing twice with Dulbecco’s phosphate-buffered saline (DPBS, PAN-Biotech) and replaced by full growth medium with or without the test substances. For scanning of the scratch wounded area, automated brightfield imaging was performed every 15 min for a total of 315 min on an epi‑fluorescence microscope (Nikon Eclipse Ti2, Nikon, Tokyo, Japan) using a 2 × CFI Plan Apochromat objective (NA = 0.1) with additional 1.5 × magnification. The microscope was further equipped with a cage incubator (Okolab, Shanghai, China) for temperature and CO_2_ control, a Zyla 4.2 sCMOS camera (Andor, Oxford Instruments, Abingdon, UK), and an x-y-stage (CMR-STG-MHIX2-motorized table, Märzhäuser Wetzlar, Wetzlar, Germany) allowing scanning of multiple stage positions in one experiment. Cage incubator preheating and CO_2_ equilibration was started 1 h prior to experiment and illumination adjustment was performed after the plate was inserted to the microscope stage. Acquisition positions were defined under the control of the NIS-Elements software package (Version 5.02.01, Nikon) with respect to the scratch in each individual well. Image analysis was carried out using *ImageJ* and the “*Wound_healing_size_tool.ijm*” plugin (Suarez-Arnedo et al., 2020). Cell front velocity was calculated as indicated:

$$cell\;front\;velocity\;\left[\mu m/h\right]=\;\frac{wound\;closure\;speed\;\left[\mu m^{2}/h\right]}{length\;of\;cell\;front\;\left[\mu m\right]\;\times \;2}$$

with wound closure speed calculated from the slope of the linear trend line (15 − 285 min) and the *length of cell front* × *2* corresponds to the length of the gap area considering two cell fronts that migrate towards each other (see also “ibidi- cells in focus”).

### Determination of intracellular reactive oxygen species

For intracellular reactive oxygen species (ROS) measurements, IPEC-J2 (4 × 10^4^ cells per well) or Caco-2 (7 × 10^4^ cells per well) cells were seeded in black 96‐well plates at and grown overnight under standard conditions. On the following day, the cells were washed twice with DPBS followed by a 20 min co-treatment with the test substances in combination with 50 µmol/L of the ROS probe 2´,7´-dichlorodihydrofluorescein diacetate (H_2_DCFDA, Sigma-Aldrich) in serum-free medium. This procedure was described previously (Heckmann et al., 2024; Wan et al., 2015). After two more washing steps with DPBS, oxidative stress was induced with 200 µmol/L 2,2´-azobis(2-methylpropionamidine) dihydrochloride (AAPH, Sigma-Aldrich) in Hank´s balanced salt solution (HBSS, PAN-Biotech). The amount of 2´,7´-dichlorodihydrofluorescein (H_2_DCF), the oxidized form of H_2_DCFDA, was determined with the POLARstar Omega microplate reader (BMG LABTECH, Ortenberg, Germany) in fluorescence mode at 485 nm excitation and 520 nm emission. The measurements were performed immediately after stressor addition and after 30, 60 and 90 min. The oxidative status over this period was summarized by calculation of the area under the curve (AUC) and normalized to the AAPH stress control.

### Detection of NO production

The Griess assay was used to determine the nitric oxide (NO) production in RAW 264.7 macrophages after stimulation with lipopolysaccharides (LPS) and co-treatment with the test substances. Secreted NO is immediately oxidized to nitrite (NO_2_^−^) molecules. In the experimental procedure, these react with sulfanilamide and N-(1-naphthyl) ethylenediamine dihydrochloride (NED) to form a colored azo compound. NO production thus correlates with the intensity of the color, measured spectrophotometrically (Sun et al., 2003). In this study, the quantification of NO production was evaluated as previously described (Heckmann et al., 2024) using the Promega Griess Reagent System according to the manufacturer’s instructions (Promega Corporation, Fitchburg, WI). In short, RAW 264.7 cells were seeded into 96-well plates (6 × 10^4^ cells per well) and grown overnight. The test substances were diluted in full growth medium with additional 250 mg/mL LPS (Sigma-Aldrich) as an activator of NO production. Cells were treated for 24 h before 50 μL of the sulfanilamide solution were added to 50 μL of each cell supernatant and incubated for 5 min at room temperature. Subsequently, 50 μL of the NED solution was added followed by additional incubation time of 5 min at room temperature. Finally, the absorbance at 548 nm was detected with the POLARstar Omega microplate reader. All results were normalized to the LPS stress control.

### NF-κB ativation analysis

To assess NF-κB activation after treatment with LPS, TNF-α, magnolol and honokiol, the HEK-Blue hTLR4 reporter cell line and its parental cell line HEK-Blue null2 were used by measuring the amount of secreted SEAP, a downstream target, as described previously (Heckmann et al., 2025a). The use of the parental cell line helped to determine the involvement of the TLR4 receptor. Briefly, 20 µL of LPS, TNF-α, magnolol and honokiol, with or without LPS and TNF-α, diluted in DMEM (supplemented with 10 % heat-inactivated FBS, 100 U/mL penicillin, 100 μg/mL streptomycin, and 100 µg/mL Normocin™) to final concentrations of 10 ng/mL LPS or TNF-α and 10 µM magnolol or 10 µM honokiol or a mixture with 5 µM of both, were added to 96-well plates. Then, 180 µL of HEK-Blue hTLR4 cells and HEK-Blue null2 cells were seeded in triplicate into the plates containing the treatment solutions at densities of 5 × 10^4^ and 7 × 10^4^ cells per well, respectively. After incubating for 24 h at 37°C, 20 µL of the cell culture supernatants were mixed with 180 µL of QUANTI-Blue (InvivoGen) solution containing the SEAP substrate. Absorbance was measured at 620 nm after 30 min of incubation at 37°C using the POLARstar Omega microplate reader. Cell viability was confirmed using the CellTiter-Glo® Cell Viability Assay (Promega Corporation, Madison, WI) according to the manufacturer´s instructions. Results were corrected for background and normalized to the stress controls.

### Statistical analysis

All statistical analyses were performed with GraphPad Prism version 11.0.0 (GraphPad Software Inc., San Diego, CA). Differences between more than two groups were examined using one-way analysis of variance (ANOVA) with Dunnett’s or Šidák’s multiple comparison test. For comparison of two groups, an unpaired t-test with Welch’s correction was applied. Differences were considered significant at ***P < 0.001, **P < 0.01 and *P < 0.05. Figures were prepared using Corel Draw version 2019 (Corel Corporation, Ottawa, ON, Canada). All box-and-whisker plots indicate the median, the replicates as single dots and the min/max as error bars.

## Results

### Wood-lignan-based feed supplement improves performance and nutrient digestibility in broilers

To evaluate the biological efficacy of the wood-lignan-based feed supplement under practical production conditions, a 35-day feeding trial was conducted in male broiler chickens. Birds received either a control diet or the same diet containing the feed supplement at 0.1 g/kg feed. Growth performance was monitored throughout the experimental period and nutrient digestibility as well as intestinal gene expression were assessed at the end of the trial to obtain a comprehensive view of the physiological effects of the feed supplement.

Growth performance data are presented in Table 4. At the start of the experiment, no differences in BW were observed between groups. On day 14, birds of the trial group showed a higher BW compared to the control group (P < 0.001), corresponding to increased daily weight gain (P < 0.001), while feed intake was not affected. FCR (P = 0.016) and EPEF (P = 0.008) were improved in the trial group during the starter phase (day 0-14).

**Table 4:** Performance parameters of broiler chickens during the different feeding phases.

Parameter	Control group	Trial group	Pooled SEM	P
	*day 0*			
Number of birds (day 0)	90	90		
BW (day 0) [g]	41.88	41.88	0.15	>1
	*day 0-14*			
Number of birds (day 14)	86	88		
BW (day 14) [g]	370.99	401.82	4.60	<0.001
Daily BW gain (day 0-14) [g]	23.51	25.71	0.32	<0.001
Cumulative feed intake (day 0-14) [g]	437.96	447.38	5.24	0.235
Daily feed intake (day 0-14) [g]	31.28	31.96	0.37	0.246
FCR (day 0-14)	1.33	1.24	0.02	0.016
EPEF (day 0-14)	169.13	202.78	7.22	0.008
	*day 15-35*			
Number of birds (day 35)	85	87		
BW (day 35) [g]	1644.33	1735.17	21.00	0.012
Daily BW gain (day 15-35) [g]	60.64	63.49	0.97	0.064
Cumulative feed intake (day 15-35) [g]	1823.32	1780.33	28.07	0.304
Daily feed intake (day 15-35) [g]	86.82	84.78	1.34	0.312
FCR (day 15-35)	1.43	1.34	0.02	0.002
EPEF (day 15-35)	449.01	471.19	13.87	0.024
	*day 0-35*			
Daily BW gain (day 0-35) [g]	45.78	48.38	0.60	0.013
Cumulative feed intake (day 0-35) [g]	2261.28	2227.71	30.73	0.458
Daily feed intake (day 0-35) [g]	64.61	63.65	0.87	0.460
FCR (day 0-35)	1.41	1.32	0.013	<0.001
EPEF (day 0-35)	306.75	356.02	10.83	0.009

*n* = 6 pens; df = 10; values represent mean; unpaired t-test.

During the grower phase (day 15-35), daily BW gain of the birds in the trial group showed a tendency to increase (P = 0.064) compared to the control group, albeit not significant. Feed intake remained unchanged and FCR (P = 0.002) and EPEF (P = 0.024) were improved in the trial group.

On day 35 BW of the animals in the trial group was higher than in the control group (P = 0.012). Over the entire experimental period (day 0-35), the wood-lignan-based supplementation resulted in a increase in daily weight gain (P = 0.013). As feed intake was not affected, feed efficiency was significantly improved, as indicated by a lower FCR (P < 0.001). EPEF was increased in the trial group compared to the control group (P = 0.009).

The digestibility of selected nutrients was assessed on day 35 using 24 birds per group. The wood-lignan-based feed supplement enhanced the AID of crude ash (P = 0.025), crude protein (P < 0.001), calcium (P < 0.001) and phosphorus (P = 0.006) compared to the control diet, as demonstrated in Table 5.

**Table 5:** Apparent ileal digestibility (AID) values calculated for the overall experimental period.

Parameter	Control group	Trial group	Pooled SEM	P
AID of crude ash [%]	43.35	45.03	0.47	0.025
AID of crude protein [%]	86.05	88.47	0.32	<0.001
AID of calcium [%]	60.02	63.09	0.36	<0.001
AID of phosphorus [%]	61.34	62.69	0.30	0.006
BW of sampled birds [g]	1648.00	1745.38	13.68	<0.001
Dry matter of ileal digesta [%]	19.39	19.14	0.53	0.747

*n* = 8 pooled ileal digesta of 3 birds each; df = 14, values represent mean; unpaired t-test;.

**Fig. 1:**
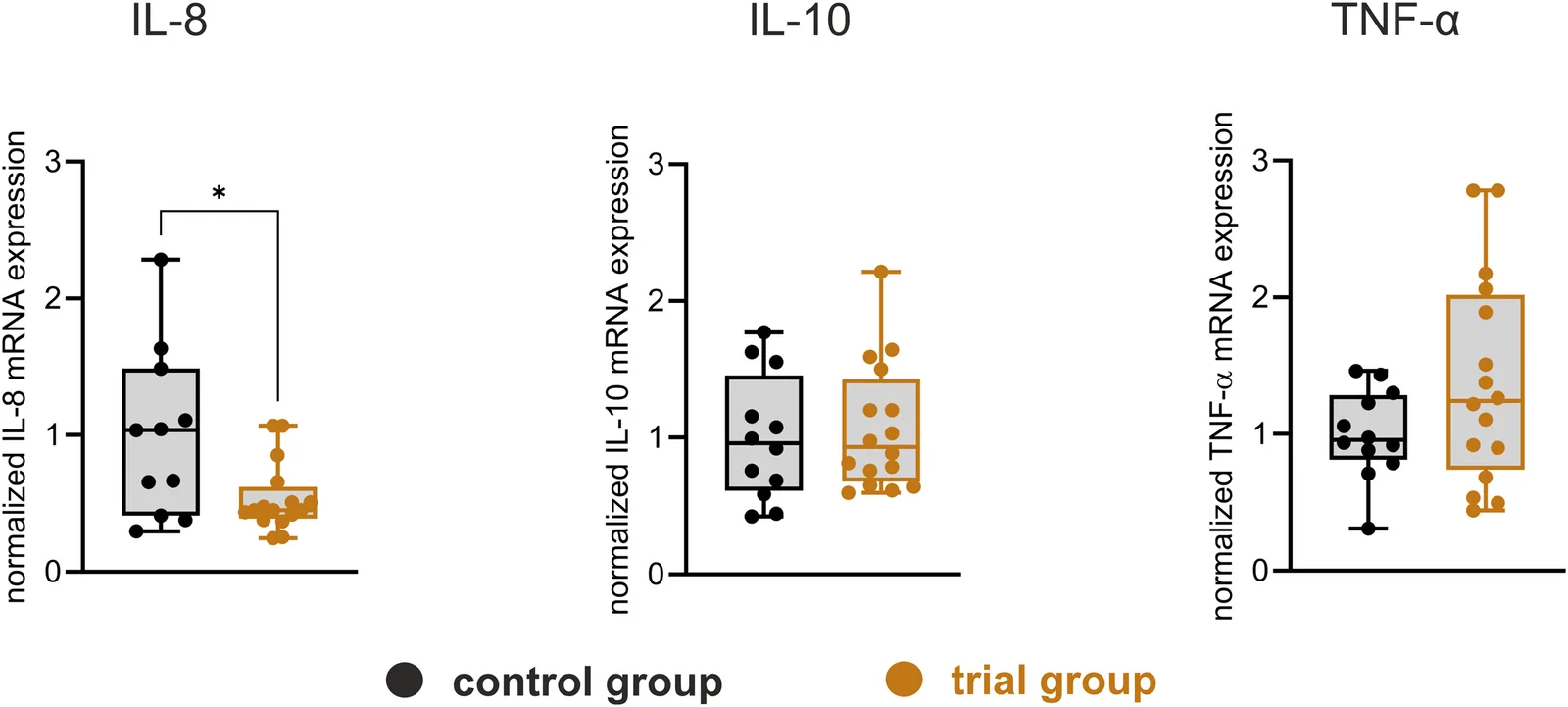
Effects of wood-lignan-based supplementation on intestinal inflammatory gene expression in broiler chickens. Relative mRNA expression of IL-8, IL-10 and TNF-α in the jejunal tissue collected at day 35 of the feeding trial (*n* = 12 in control group and *n* = 16 in trial group). Gene expression was normalized to the reference genes and expressed relative to the control group. *P < 0.05, compared to the control group (unpaired t-test).

On day 35, jejunal tissue samples from 12 birds per treatment were collected to determine cytokine gene expression. As shown in Fig. 1, the wood-lignan-based supplementation resulted in a downregulation of IL-8 mRNA expression (P = 0.017) compared to the control diet. No treatment effects were observed for TNF-α and IL-10 expression. TNF-α expression showed a non-significant, numerically higher mean in the trial group, with high inter-individual variability. IL-6 transcripts were not detectable in either group.

### Lignans are transported across the intestinal epithelium in vitro

To further elucidate potential mechanisms underlying the effects observed in the broiler trial, complementary in vitro experiments were conducted using intestinal epithelial and immune cell models. Since our previous study hypothesized that the main lignans present in the wood-based feed supplement, magnolol and honokiol, mediate its beneficial effects, this assumption was analyzed more thoroughly in the present work (Heckmann et al., 2022).

**Fig. 2:**
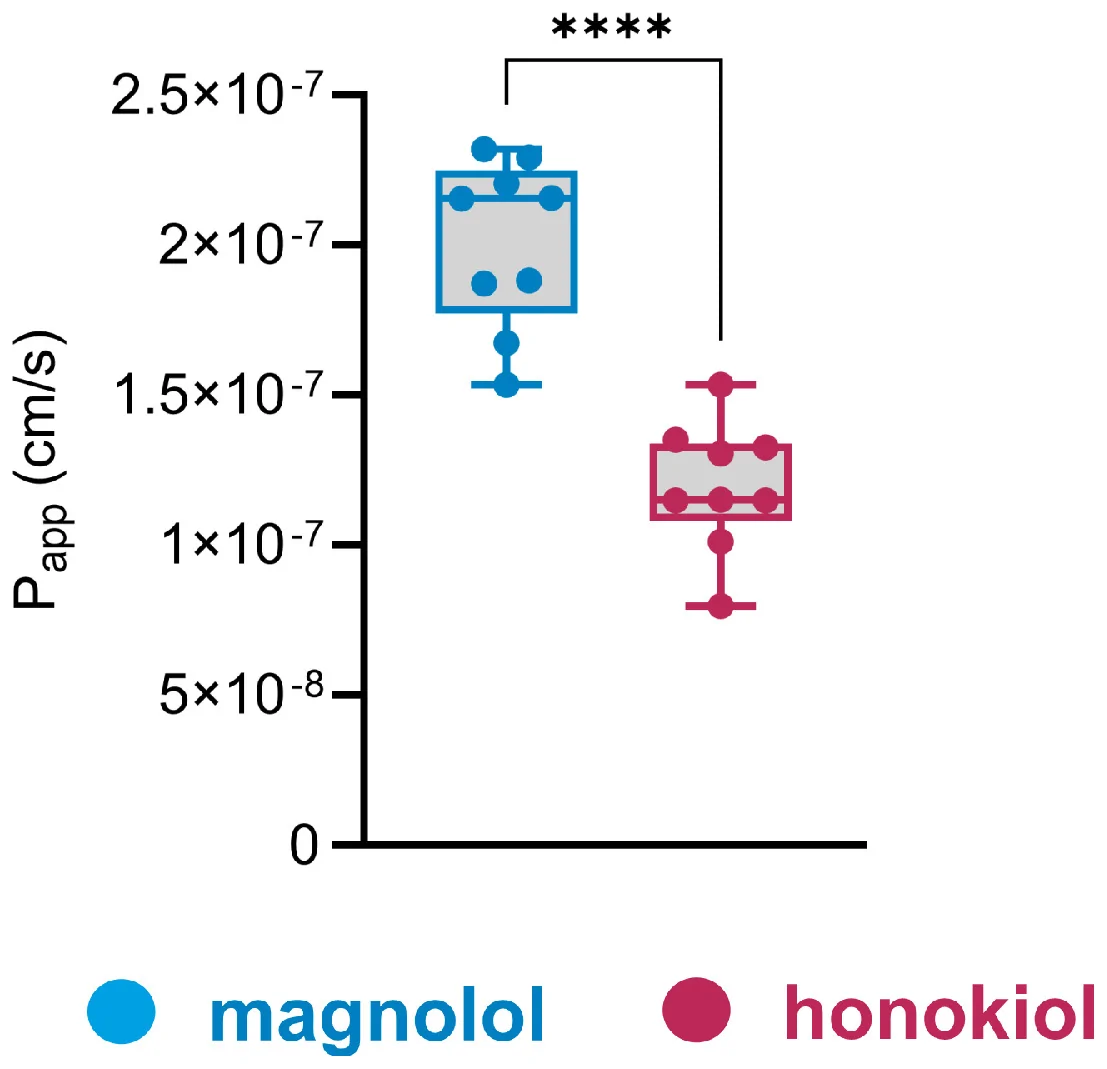
Transport of magnolol and honokiol across a Caco-2 intestinal epithelial monolayer. Compounds were applied apically and apparent permeability coefficients (P_app_) were calculated after the determination of the concentration in the basolateral compartment following 4 h of incubation (*n* = 9, 3 independent experiments). ***P < 0.001 (unpaired t-test).

Before investigating magnolol and honokiol in additional cell models, their intestinal transport was assessed using the transwell insert-based Caco-2 monolayer model. Each compound was applied apically at 100 µM and concentrations in the basolateral compartment were measured after 4 h to calculate the apparent permeability coefficient (P_app_). As shown in Fig. 2, both magnolol and honokiol were transported across the Caco-2 intestinal monolayer, with magnolol exhibiting a significantly higher P_app_ value (2.01 × 10^−7^ cm/s), compared to honokiol (1.20 × 10^−7^ cm/s).

### Lignans promote wound healing and reduce oxidative stress in intestinal cells

**Fig. 3:**
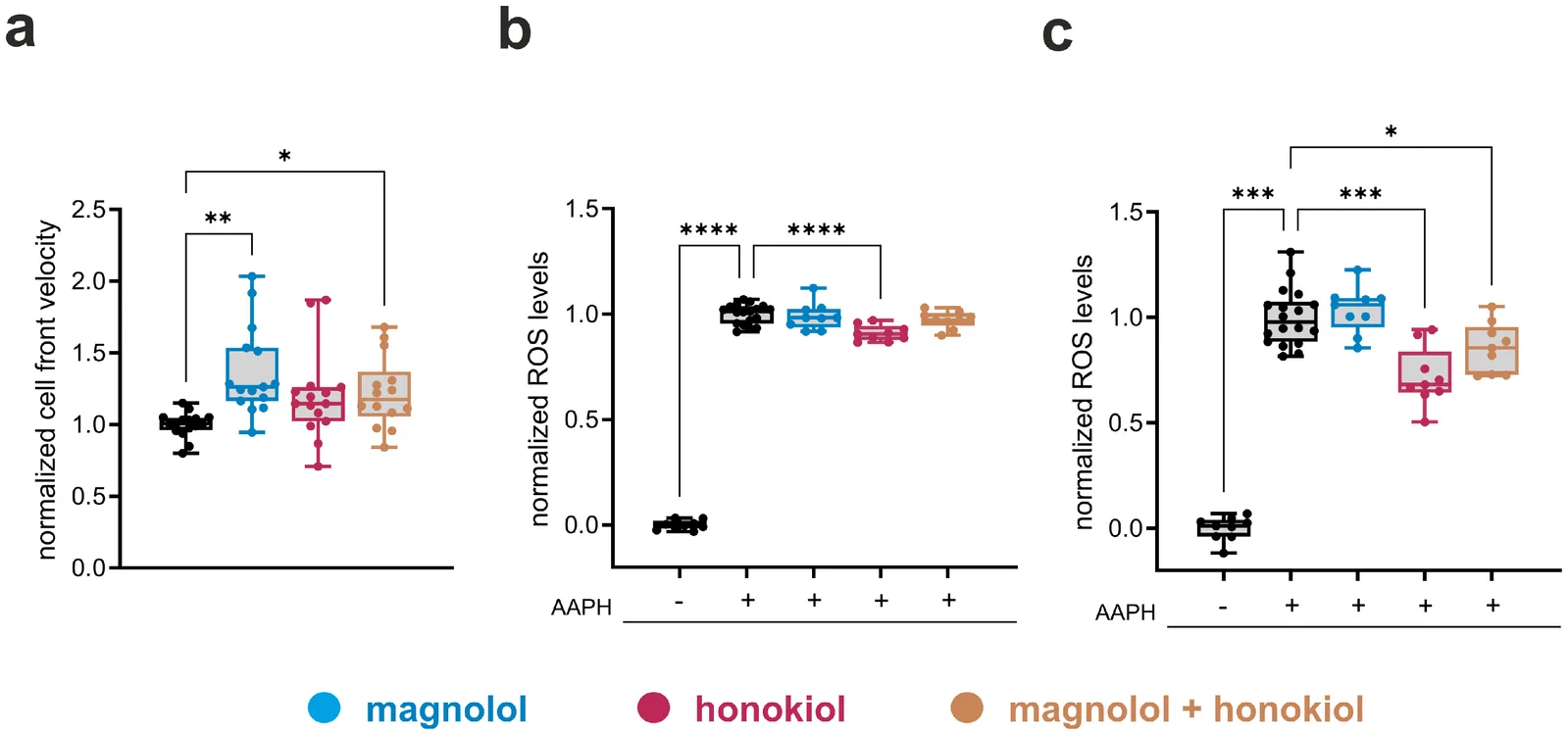
Effects of magnolol and honokiol on cell migration and ROS production in intestinal cells. (**a**) Cell front velocity of the scratch closure, normalized to the control. The IPEC-J2 cell monolayer grown in 24-well plates was scratched with a pipette tip. Magnolol (5 µM), honokiol (5 µM) and their combination (5 µM each) were added to the cells and brightfield images were taken every 15 min (*n* = 14-16, *4* independent experiments). (**b**) ROS production in IPEC-J2 and (**c**) Caco-2 cells treated with (**b**) 5 µM or (**c**) 10 µM magnolol and honokiol, and their combination (5 µM each) following oxidative stress induction using AAPH. AUC of fluorescence in the stress phase represents ROS levels (*n* = 9-18, 3 independent experiments). ***P < 0.001, **P < 0.01, *P < 0.05, compared to untreated control (**a**) or oxidative stress control (**b, c**) (one-way ANOVA).

The impact of magnolol and honokiol on epithelial restitution was assessed using a scratch assay in IPEC-J2 cells. Confluent monolayers were mechanically disrupted with a pipette tip and subsequently treated with pure compounds. Wound closure was monitored at 15-min intervals by automated brightfield microscopy. Cell front velocity, defined as the rate of epithelial migration into the injured area, was used as a quantitative measure of cell migration. As shown in Fig. 3a, magnolol at 5 µM increased wound closure speed compared to the untreated control (P = 0.001). Similarly, the combination of magnolol and honokiol (5 mM each) enhanced epithelial migration (P = 0.016), whereas honokiol at 5 µM caused no significant change. These results indicate that magnolol plays a prominent role in promoting epithelial repair after mechanical injury.

Because impaired wound healing is closely linked to oxidative stress and elevated ROS levels (Kurahashi and Fujii, 2015), we next analyzed the ability of magnolol and honokiol to counteract oxidative stress in intestinal cells. Fig. 3b and Fig. 3c illustrate the results of the ROS generation under oxidative stress in IPEC-J2 and Caco-2 cells, respectively. The increase of intracellular ROS level following oxidative stress induction with the free-radical-generating compound AAPH could be affected by the pretreatment with the tested compounds. In IPEC-J2 cells, honokiol at 5 µM reduced the ROS production upon oxidative stress by 9.06 % (P < 0.001). In contrast, magnolol at 5 µM and the lignan combination at 5 µM each did not have an influence. In Caco-2 cells, honokiol at 10 µM and the combination of 5 µM honokiol and 5 µM magnolol led to a decrease in ROS levels by 28.17 % (P < 0.001) and 14.36 % (P = 0.041), respectively. A higher lignan concentration was applied to Caco-2 cells because preliminary cytotoxicity testing revealed greater tolerance of this cell line compared to IPEC-J2 cells, which exhibited increased sensitivity to higher lignan concentrations.

These findings indicate that honokiol demonstrates significant antioxidant activity in vitro, as evidenced by a reduction in intracellular ROS production in two intestinal cell lines. In contrast, magnolol showed limited antioxidant effects under the tested conditions but more strongly enhanced epithelial cell migration upon mechanical stress induction. These data suggest complementary modes of action of the two lignans, with magnolol primarily supporting epithelial restitution and honokiol contributing to cellular redox homeostasis.

### Lignans reduce in vitro pro-inflammatory responses through inhibition of NF-κB signaling

**Fig. 4:**
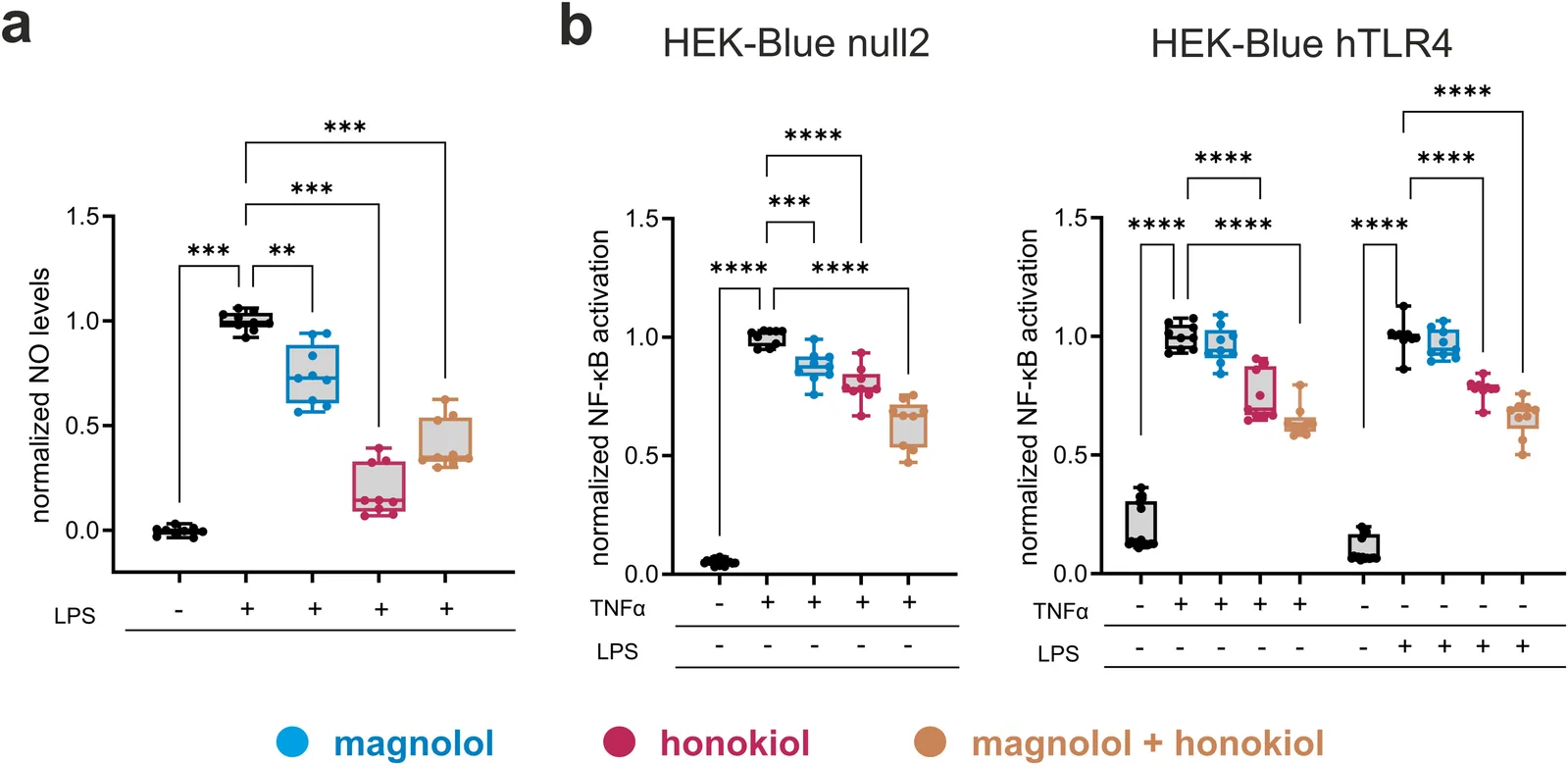
Effects of magnolol and honokiol on NO levels and NF-κB activation in vitro. (**a**) NO production in LPS-activated RAW 264.7 macrophages following co-treatment with magnolol (10 µM), honokiol (10 µM) or their combination (5 µM each) for 24 h, normalized to the stress control (*n* = 9, 3 independent experiments). (**b**) NF-κB activation in non-TLR4-expressing HEK-Blue null2 cells and TLR4-expressing HEK-Blue hTLR4 cells following a 24 h treatment with TNF-α, LPS, magnolol (10 µM), honokiol (10 µM) or their combination (5 µM each), normalized to the stress control (*n* = 9-18, 3 independent experiments). ***P < 0.001, **P < 0.01, compared to LPS- or TNF-α stress control, respectively (one-way ANOVA).

To assess the anti-inflammatory potential of magnolol and honokiol, LPS-activated RAW 264.7 macrophages were used as an inflammation model, with NO production serving as a functional readout of activation. As shown in Fig. 4a, treatment with magnolol (10 µM), honokiol (10 µM) or a combination of both (5 µM each) significantly reduced NO levels (P < 0.001). Honokiol exhibited the most pronounced effect, reducing the secretion by 80.82 %.

Next, we investigated whether these observed effects involve modulation of NF-κB signaling and whether this occurs via TLR4-dependent or TLR4-independent pathways. Distinguishing between TLR4-dependent and independent NF-κB activation is important to determine whether the compounds interfere with upstream pathogen recognition mechanisms or act on downstream inflammatory signaling pathways. HEK-Blue hTLR4 reporter cells, which stably express TLR4 and SEAP, and the parental HEK-Blue null2 cells expressing only SEAP, were used for this analysis. SEAP secretion directly reflects NF-κB activation. As illustrated in Fig. 4b, treatment with 10 µM honokiol and the combination of magnolol and honokiol (5 µM each) inhibited both TNF-α- (P < 0.001) and LPS-induced (P < 0.001) NF-κB activation in HEK-Blue hTLR4 cells, as well as TNF-α-induced NF-κB activation in HEK-Blue null2 cells (P < 0.001). In contrast, magnolol at 10 µM only significantly reduced TNF-α-induced NF-κB activation in HEK-Blue null2 cells (P < 0.001). These results suggest that honokiol alone or in combination with magnolol inhibits both TLR4-dependent and TLR4-independent NF-κB activation, whereas magnolol alone shows only a slight tendency to reduce active NF-κB levels.

## Discussion

Maintenance of intestinal health is considered essential for optimal growth performance, nutrient utilization, and resilience against enteric challenges in commercial broiler production. The present study investigated the effects of a wood-derived lignan feed supplement containing magnolol and honokiol on intestinal health and metabolic efficiency using a comprehensive in vivo and in vitro approach. In the broiler feeding trial, dietary supplementation improved growth performance and nutrient utilization, as reflected by enhanced BW gain and feed efficiency.

While several studies have demonstrated beneficial effects of magnolol and honokiol on growth performance, antioxidant capacity and intestinal health in broiler chickens, information regarding their effects on nutrient digestibility remains scarce. Previous work with magnolol has reported improvements in average daily gain and FCR, as well as enhanced antioxidant capacity and modulation of gut microbiota composition in broilers (Xie et al., 2022). Similarly, dietary honokiol supplementation has been shown to increase BW gain, reduce FCR and improve intestinal histomorphology, with effects attributed to enhanced antioxidant enzyme expression and altered cecal microbial community structure (Du et al., 2024). However, these studies did not include direct measurements of nutrient digestibility or mineral utilization. Instead, improved performance was mainly attributed to enhanced intestinal health and metabolic function based on indirect indicators such as histomorphology and microbial shifts. Similarly, studies using Magnolia bark extracts reported alterations in intestinal metabolic profiles suggesting improved nutrient processing, but without quantifying digestibility coefficients (Park et al., 2020). The present study extends this knowledge by directly demonstrating that lignan supplementation improves apparent ileal digestibility of crude ash, crude protein, calcium and phosphorus, supporting a functional improvement in nutrient utilization efficiency. Although apparent ileal digestibility of crude ash should be interpreted with caution because crude ash represents a heterogenous mixture of minerals and may be influenced by endogenous mineral secretions, the observed increase was consistent with the improvements in calcium and phosphorus digestibility. Therefore, the crude ash results are considered supportive of the overall mineral digestibility response rather than an independent biological outcome. Additionally, the present study did not directly assess digestive enzyme activity or gut microbiota composition, which may also contribute to the observed changes in nutrient utilization.

At the molecular level, jejunal gene expression analysis revealed significant downregulation of IL-8, whereas no significant differences were observed for TNF-α or IL-10. IL-6 expression was below the detection limit in the jejunal samples. IL-8 is a key chemokine involved in the recruitment of immune cells and the initiation of local inflammatory responses in the avian intestine (Kogut, 2000). IL-8 has previously been proposed as a potential biomarker for gut barrier health in broiler chickens, as its expression is closely associated with gut barrier failure and mucosal inflammatory activation (Chen et al., 2015). In the present study, the observed downregulation of IL-8 at day 35 may therefore reflect a reduced inflammatory tone in the jejunum, which could be linked to improved intestinal functionality and nutrient utilization. This interpretation is consistent with evidence that reduction of intestinal IL-8 expression in broilers is associated with a more balanced immune response and improved mucosal homeostasis (Rodríguez et al., 2023). In contrast, the lack of differences in TNF-α and IL-10 between the groups at day 35 of the trial suggests that the wood-lignan-based supplement did not markedly affect broader pro- or anti-inflammatory cytokine balance under the present experimental conditions. This interpretation is further supported by the lack of detectable IL-6 expression, a classical acute-phase and pro-inflammatory cytokine typically upregulated during pathogen challenge and suppressed to baseline levels in animals without active mucosal inflammation, indicating a low basal inflammatory status of the birds (Oakley and Kogut, 2016). However, as cytokine expression was assessed at a single time point at the end of the trial, temporal dynamics of intestinal immune modulation cannot be excluded, and corresponding protein-level changes were not assessed. Thus, the present data support a selective alteration in jejunal IL-8 expression rather than a generalized anti-inflammatory effect of the supplement.

The practical interpretation of the feeding trial should consider that, although significant improvements in performance and digestibility were observed under the present conditions, further studies under different stocking densities, flock sizes and commercial production settings are needed to confirm their reproducibility. Moreover, as only a single inclusion level was tested, a dose-dependent response could not be established and the possibility that some of the observed differences reflect pen-level variation cannot be excluded.

To further investigate biological properties of the two lignans contained in the feed supplement, magnolol and honokiol were investigated individually in intestinal epithelial and immune cell models. This approach was based on our previous findings where we demonstrated that the wood-lignan-based feed supplement reduces IL-8, IL-6 and TNF-α expression in LPS-activated THP-1 macrophages (Heckmann et al., 2022). These results suggested that the in vitro anti-inflammatory effects of the supplement may be driven, at least in part, by its lignan components, although their individual contributions and mode of action had not yet been clarified.

As an initial step, the intestinal transport of magnolol and honokiol was assessed using the Caco-2 model to evaluate their intestinal bioavailability. Both compounds were able to cross the epithelial monolayer, indicating their potential to be absorbed following oral administration. This is in line with a previous study demonstrating that magnolol can cross Caco-2 monolayers predominantly via a combination of passive diffusion, carrier-mediated and efflux transport mechanisms, resulting in a measurable apparent permeability coefficient (Wu et al., 2013). Notably, we observed that magnolol exhibited higher apparent permeability than honokiol, suggesting differences in intestinal transport characteristics or stability, which may influence their bioactivity. However, apparent permeability values derived from the Caco-2 assay should be interpreted as relative indicators of intestinal transport potential rather than a direct predictor of in vivo bioavailability, given the well-established limitations of this system (Press and Di Grandi, 2008). Intestinal microbial metabolism of lignans as well as possible enterohepatic recirculation have been described in the literature but are not reflected in this model (Bolvig et al., 2016; Hattori et al., 1986).

The mechanistic in vitro data of the present study demonstrate complementary functional roles of the two compounds. Magnolol primarily promoted epithelial restitution, as shown by enhanced wound closure in IPEC-J2 cells, indicating a direct effect on epithelial migration and repair processes following mechanical injury. The IPEC-J2 cell line is a non-transformed line derived from jejunal crypts of neonatal pigs and is widely used in wound healing assays due to its morphological functional similarity to small intestinal epithelia in vivo (Boger et al., 2023). Previous reports documented reduced inflammatory damage and improved tissue integrity following magnolol treatment, but the underlying mechanisms were primarily attributed to NF-κB and p38 mitogen-activated protein kinase (MAPK) modulation rather than epithelial migration per se (Lee et al., 2005; Tse et al., 2007). In contrast to magnolol, honokiol exhibited stronger antioxidant and anti-inflammatory properties, as evidenced by reduced ROS formation in intestinal epithelial cells and profound inhibition of NO production in LPS-activated macrophages. These findings suggest that honokiol predominantly contributes to the regulation of cellular redox balance and inflammatory activation, processes associated with growth performance, feed efficiency, intestinal morphology, nutrient absorption, and resilience against enteric disorders in broiler production (Tellez-Isaias et al., 2023). Importantly, epithelial migration, ROS production and NO production were not assessed in the broiler trial. Therefore, these observations cannot be directly linked to the improved performance or digestibility responses but provide plausible cellular mechanisms that could contribute to biological activity of the feed supplement.

At the signaling level, both lignans inhibited NF-κB activation in vitro, although with distinct potency and pathway specificity. Honokiol has been shown to inhibit LPS-induced NF-κB activation and downstream production of TNF-α and NO in macrophages, acting upstream through suppression of extracellular-signal regulated kinase (ERK)1/2, jun N-terminal kinase (JNK)1/2 and p38 MAPK phosphorylation as well as protein kinase C-α membrane translocation (Chao et al., 2010). This broad inhibitory capacity is consistent with its ample suppression of both TLR4-dependent and -independent NF-κB activation observed in the HEK-Blue reporter system in the present study. Honokiol has further been demonstrated to suppress TNF-α-stimulated NF-κB activation through inhibition of upstream IκB kinase activity, thereby preventing IκBα degradation and nuclear translocation of p65 subunit, and downstream repression of NF-κB-regulated gene products including IL-8 (Tse et al., 2005). Magnolol, in contrast, showed weaker and more selective effects, primarily affecting TNF-α-induced NF-κB activation, which aligns with its reported modulation of NF-κB and MAPK pathways in macrophage models (Lee et al., 2005; Tse et al., 2007). Collectively, these findings indicate that both lignans are capable of modulating inflammatory processes relevant to intestinal function and homeostasis in cellular models.

Although magnolol and honokiol represent the major lignan constituents of the feed supplement and were investigated individually in the present in vitro experiments, the contribution of other components of the supplement, including zinc, cannot be fully excluded. Consequently, the in vitro findings should be considered as providing potential explanations for, rather than direct mechanistic confirmation of, the responses observed in supplemented broilers.

Taken together, supplementation with the wood-derived lignan feed supplement improved growth performance and selected apparent ileal digestibility outcomes in broilers under the conditions of the present study and was associated with reduced jejunal IL-8 expression at day 35. The in vitro findings further showed that magnolol and honokiol exert distinct effects on epithelial restitution, oxidative stress and inflammatory signaling. These cellular responses provide plausible mechanistic hypotheses for the biological activity of the supplement, but their relevance to the in vivo responses and the contribution of individual supplement components require further investigation.

## Ethics declarations

All procedures performed in the broiler trial were approved by the local state office for occupational health and technical safety (Landesamt für Gesundheit und Soziales, Berlin, Germany; license number STN 035/24) and conducted in accordance with the German Animal Welfare Act, European Directive 2010/63/EU and Commission Recommendation 2007/526/EC. Animal health and welfare were monitored throughout the study.

### Consent for publication

All of the authors have approved the final version of the manuscript and agreed with this submission to *Poultry Science*.

## Competing interests

The authors declare the following financial interests/personal relationships which may be considered as potential competing interests: NN reports a relationship with agromed Austria GmbH as inventor of the tested feed supplement. NN contributed to the study concept, including the selection of the test product, inclusion level and study objectives. The detailed experimental methodology, animal trial, sample collection, laboratory analyses, data collection and statistical evaluation were independently designed and performed by the university research team. agromed Austria GmbH had no influence on the decision to report the research. The other authors declare that they have no competing interests.

## Funding

The financial support by the Austrian Federal Ministry of Economy, Energy and Tourism, the 10.13039/100010132National Foundation for Research, Technology and Development and the 10.13039/501100006012Christian Doppler Research Association is gratefully acknowledged. This research was further funded by a research project of the Austrian Competence Centre for Feed and Food Quality, Safety and Innovation FFoQSI. The COMET competence centre FFoQSI is funded by the Austrian federal ministries BMWET, BMIMI and the Austrian provinces Lower Austria, Upper Austria and Vienna within the scope of COMET - Competence Centers for Excellent Technologies. The programme COMET is managed by the Austrian Research Promotion Agency FFG.

## Author Contribution

MH and VS contributed to the conceptualization and design of the study, methodology, investigation, data curation and manuscript preparations. BS contributed to the methodology, investigation and data curation. NN contributed to the conceptualization of the study and manuscript preparation. KM contributed to the conceptualization, methodology, investigation and data curation. JW contributed to the critical review of the manuscript and funding acquisition. BB-L contributed to the conceptualization and methodology of the study, data curation, manuscript preparation, supervision, project administration and funding acquisition. All authors critically reviewed the manuscript for important intellectual content, reviewed and approved the final version of the manuscript and agree to be personally and publicly accountable for all aspects of the work.

## CRediT authorship contribution statement

**Mara Heckmann:** Conceptualization, Methodology, Investigation, Data curation, Writing – original draft, Writing – review & editing. **Verena Stadlbauer:** Conceptualization, Methodology, Investigation, Data curation, Writing – original draft, Writing – review & editing. **Bettina Schwarzinger:** Methodology, Investigation, Data curation. **Nina Neufeld:** Conceptualization, Writing – original draft, Writing – review & editing. **Klaus Männer:** Conceptualization, Methodology, Investigation, Data curation. **Julian Weghuber:** Writing – review & editing, Funding acquisition. **Bernhard Blank-Landeshammer:** Conceptualization, Methodology, Data curation, Writing – original draft, Writing – review & editing, Supervision, Project administration, Funding acquisition.

## Disclosures

The authors declare the following financial interests/personal relationships which may be considered as potential competing interests: Nina Neufeld reports a relationship with agromed Austria GmbH that includes: consulting or advisory. If there are other authors, they declare that they have no known competing financial interests or personal relationships that could have appeared to influence the work reported in this paper.

## Data Availability

Data are available from the corresponding authors upon reasonable request.
